# A Biofilm Pocket Model to Evaluate Different Non-Surgical Periodontal Treatment Modalities in Terms of Biofilm Removal and Reformation, Surface Alterations and Attachment of Periodontal Ligament Fibroblasts

**DOI:** 10.1371/journal.pone.0131056

**Published:** 2015-06-29

**Authors:** Tobias T. Hägi, Sabrina Klemensberger, Riccarda Bereiter, Sandor Nietzsche, Raluca Cosgarea, Simon Flury, Adrian Lussi, Anton Sculean, Sigrun Eick

**Affiliations:** 1 Department of Periodontology, School of Dental Medicine, University of Bern, Bern, Switzerland; 2 Centre of Electron Microscopy, University Hospital of Jena, Jena, Germany; 3 Department of Prosthetic Dentistry, University of Cluj-Napoca, Cluj-Napoca, Romania; 4 Department of Periodontology, Philips University, Marburg, Germany; 5 Department of Preventive, Restorative and Pediatric Dentistry, University of Bern, Bern, Switzerland; The University of Adelaide, AUSTRALIA

## Abstract

**Background and Aim:**

There is a lack of suitable in vitro models to evaluate various treatment modalities intending to remove subgingival bacterial biofilm. Consequently, the aims of this in vitro-study were: a) to establish a pocket model enabling mechanical removal of biofilm and b) to evaluate repeated non-surgical periodontal treatment with respect to biofilm removal and reformation, surface alterations, tooth hard-substance-loss, and attachment of periodontal ligament (PDL) fibroblasts.

**Material and Methods:**

Standardized human dentin specimens were colonized by multi-species biofilms for 3.5 days and subsequently placed into artificially created pockets. Non-surgical periodontal treatment was performed as follows: a) hand-instrumentation with curettes (CUR), b) ultrasonication (US), c) subgingival air-polishing using erythritol (EAP) and d) subgingival air-polishing using erythritol combined with chlorhexidine digluconate (EAP-CHX). The reduction and recolonization of bacterial counts, surface roughness (Ra and Rz), the caused tooth substance-loss (thickness) as well as the attachment of PDL fibroblasts were evaluated and statistically analyzed by means of ANOVA with Post-Hoc LSD.

**Results:**

After 5 treatments, bacterial reduction in biofilms was highest when applying EAP-CHX (4 log10). The lowest reduction was found after CUR (2 log10). Additionally, substance-loss was the highest when using CUR (128±40 µm) in comparison with US (14±12 µm), EAP (6±7 µm) and EAP-CHX (11±10) µm). Surface was roughened when using CUR and US. Surfaces exposed to US and to EAP attracted the highest numbers of PDL fibroblasts.

**Conclusion:**

The established biofilm model simulating a periodontal pocket combined with interchangeable placements of test specimens with multi-species biofilms enables the evaluation of different non-surgical treatment modalities on biofilm removal and surface alterations. Compared to hand instrumentation the application of ultrasonication and of air-polishing with erythritol prevents from substance-loss and results in a smooth surface with nearly no residual biofilm that promotes the reattachment of PDL fibroblasts.

## Introduction

Biofilms are naturally occurring accumulations of microorganisms, that are embedded in an extracellular polymeric matrix and adherent to biologic or non-biologic surfaces [1]. Microorganisms communicate via “quorum sensing”, they control virulence, motility, metabolism, production of antibiotics, exchange of genes, formation of biofilm [1]. Antimicrobials have no or limited efficacy against microorganisms in biofilms. They penetrate slowly or incompletely through the biofilm matrix [2]. Microorganisms respond to heterogeneous environment of biofilms by gene transfer leading to higher resistance and higher virulence; subpopulations consist of dormant and metabolically less active «persister» bacteria [2]. Consequently, mechanical removal of biofilms is still of major importance in any biofilm-associated disease.

One of the most prevalent biofilm-associated diseases is periodontitis, an inflammatory condition affecting the hard and soft tissue surrounding teeth. If left untreated, periodontitis leads to tooth loss. Nowadays, pathogenesis of periodontitis is thought as a dysbiosis of biofilm associated with deviated bacterial function [3] followed by an immuno-inflammatory host-mediated destruction of bone and connective tissues [4]. In this process *Porphyromonas gingivalis*, a gram-negative bacterium, was postulated being a key-stone pathogen [5]. A few other bacteria, *e*.*g*. *Tannerella forsythia*, *Treponema denticola* [6], and *Aggregatibacter actinomycetemcomitans* [7] may contribute. Moreover, bacteria within subgingival biofilms may serve as a link to systemic diseases, e.g. rheumatoid arthritis [8].

The primary goal in cause-related periodontitis treatment is to remove hard and soft bacterial deposits which should result in a smooth and biocompatible root surface to minimize bacterial adhesion and to facilitate fibroblast attachment. Since in vitro bacterial compounds (i.e. *P*. *gingivalis* lipopolysaccharide) stimulate expression of proinflammatory chemokine interleukin-8 in PDL fibroblasts [9] it can be anticipated that a minimized bacterial adhesion might prevent or reduce the inflammatory reaction of periodontal ligament (PDL) fibroblasts. Scaling and root planing (SRP), which includes the mechanical removal of biofilms, is an effective causative method for infection control in the treatment of periodontal disease [10]. A supportive maintenance program after initial therapy prevents the incidence of caries and further periodontal breakdown [11]. Based on the individual risk for disease progression, patients are seen for periodontal maintenance therapy at varying intervals ranging from three to 12 months [12]. Apart from repeated instruction and remotivation for the patients’ plaque control, the removal of subgingival bacterial deposits is one major therapeutical goal to maintain periodontal stability. Despite the introduction of machine driven sonic and ultrasonic scalers, the use of hand instruments (i.e. conventional Gracey curettes) is still widely spread in dental practice. Comparisons between hand instruments and sonic / ultrasonic scalers did not show a clear advantage for the machine driven instruments in terms of the clinical results [13,14]. On the other hand, the use of hand instruments may result in higher tooth hard-substance-loss [15,16]. Therefore, in order to prevent extensive tooth hard-substance-loss during periodontal maintenance, treatment modalities that combine both, a complete removal of the biofilm and a minimal tooth hard-substance-loss, should be preferred.

As a novel approach, air-polishing using low abrasive glycine powder was indicated to remove subgingival biofilm, and its abrasivity was demonstrated to be approximately 80% lower than that of the previously used bicarbonate air-polishing powder [17]. Clinical studies of patients in periodontal maintenance demonstrated comparable clinical results as when using hand instruments [18,19,20]. However, the patients’ acceptance was better and the treatment time was shorter [19]. Inconsistent microbiological results were reported, partially demonstrating a higher decrease of the total viable counts and of *P*. *gingivalis* [18], whereas in the study by Moëne et al. [19] bacterial counts were pronouncedly reduced in the group using hand instruments.

Apart from glycine, erythritol was recently introduced as a low-abrasive air-polishing powder [21]. Erythritol is a polyol and used in food industry as an artificial sweetener. Its physical properties were shown to be comparable to glycine [22]. Moreover, when used during supportive periodontal therapy, similar results in terms of clinical and microbiological outcome were demonstrated in comparison with hand instrumentation [22]. As of late, supplementation of the erythritol powder with chlorhexidine digluconate as an antimicrobial was available [23].

The lack of suitable in vitro models to evaluate traditional and newly developed treatment modalities for non-surgical treatment of periodontitis made us establish a multi-species biofilm pocket model to perform in vitro evaluation of repeated mechanical removal of biofilms. Thus, the aims of the present in vitro-study were: a) to establish a biofilm model which mimics the subgingival environment of a periodontal pocket and b) to use for the first time the new model to evaluate the most frequently used methods for biofilm removal ((i.e. hand instrumentation using curettes (CUR) and ultrasonication (US)) with recently developed ones (air-polishing using erythritol only (EAP) or supplemented with chlorhexidine (EAP-CHX)) in terms of biofilm removal and reformation, caused surface alterations, tooth hard-substance-loss, and the attachment of periodontal ligament fibroblasts.

## Material and Methods

### Preparation of specimens

**Fig 1:**
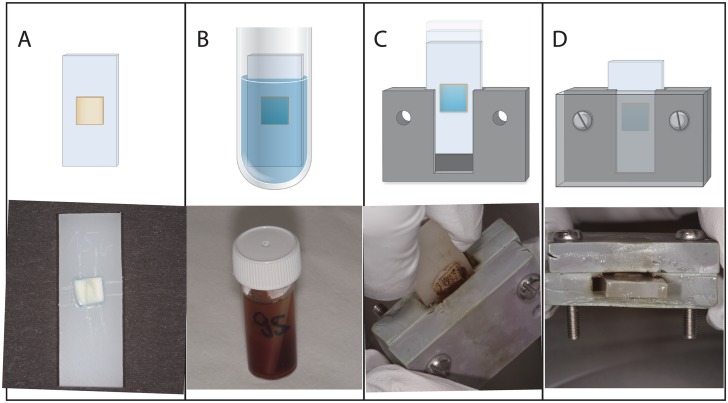
A-D. Dentin Specimen, Biofilm Formation, and Pocket Model. Schematic illustration and corresponding photographs of the biofilm formation on dentin specimen (A+B) and the insertion of the dentin specimen into the artificial pocket model (C+D).

Teeth extracted for periodontal reasons were collected as anonymous by-products of regular treatment of patients giving their written informed consent for the use in an in vitro experiment which was approved by the Ethics Committee of the University of Bern. After extraction, teeth were stored in 60% ethanol for disinfection and processed within 2–3 weeks. After crown removal, dentin slices of the buccal side of the roots of canines and premolars were cut with a diamond saw to dimensions of 4 mm x 4 mm and to a thickness of approximately 1 mm. Dentin specimens were then adhesively fixed onto plastic specimen holders using a dental dentin adhesive system (OptiBond FL, Kerr, Scafati, Italy). Surface properties of the buccal side of the dentin specimens were standardized by grinding the dentin specimens with silicon carbide papers of #2400 grit size, corresponding to an abrasive particle size of 6.5 μm (Struers A/S, Ballerup, Denmark) and in a manner to be exposed approximately 500 μm over the plastic specimen holder (Fig 1A).

### Biofilm formation on specimens

All specimens were colonized with a biofilm as recently described [24]. For biofilm formation, a multiple species mixture consisting of 12 bacterial strains (*Streptococcus gordonii* ATCC 10558, *Actinomyces naeslundii* ATCC 12104, *Fusobacterium nucleatum* ATCC 25586, *Campylobacter rectus* ATCC 33238, *Eubacterium nodatum* ATCC 33099, *Eikenella corrodens* ATCC 23834, *Parvimonas micra* ATCC 33270, *Prevotella intermedia* ATCC 25611, *Porphyromonas gingivalis* ATCC 33277, *Tannerella forsythia* ATCC 43037, *Treponema denticola* ATCC 35405, *Aggregatibacter actinomycetemcomitans* Y4) was prepared. Before the experiment, all strains (except for *T*. *denticola* ATCC 35405) were precultivated on Schaedler agar plates (Oxoid, Basingstoke, UK) with 5% sheep blood in an anaerobic atmosphere or with 5% CO_2_ (*A*. *actinomycetemcomitans* Y4 and *S*. *gordonii* ATCC 10558). *T*. *denticola* ATCC 35405 was maintained in modified mycoplasma broth (BD, Franklin Lake, NJ) added by 1 mg/ml glucose, 400 μg/ml niacinamide, 150 μg/ml spermine tetrahydrochloride, 20 μg/ml Na isobutyrate enriched with 1 g/ml cysteine and 5 μg/ml cocarboxylase in anaerobic conditions.

First the specimens were dipped into 25% inactivated human serum for 10 min and thereafter placed into tubes. Then, bacterial suspension was added for 3.5 days (Fig 1B).

### Pocket model

An artificial pocket model was established using two forms of polyether (Impregum, 3M ESPE, Seefeld, Germany) resulting in an artificial pocket, which could be opened for specimen insertion and removal, thus not destroying the established biofilm (Fig 1C+1D).

### Instrumentation

**Fig 2:**
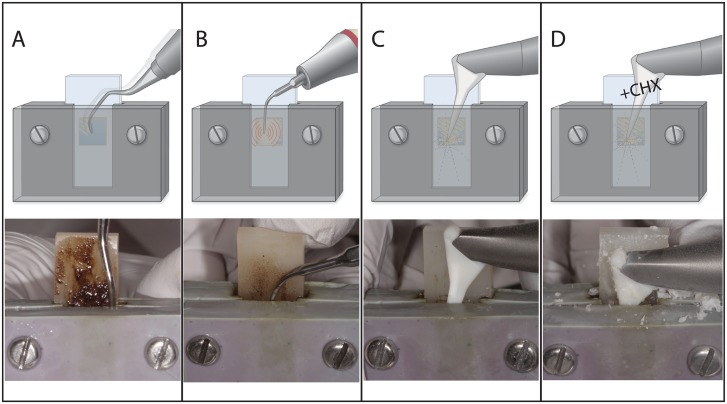
A-D. Dentin Specimen, Biofilm Formation, and Pocket Model. Schematic illustration and corresponding photographs of the four treatment modalities hand instrumentation (A), ultrasonication (B), erythritol air-polishing (EAP; C), and EAP with chlorhexidine digluconate (D).

After 3.5 days biofilm formation, the specimens were treated by mechanical debridement in the artificial pocket using CUR (Fig 2A; 10 strokes at average working pressure using 11GC12 Gracey curettes, Deppeler SA, Rolle, Switzerland), US (Fig 2B; mid water and power setting for 10 s; Air-Flow Master Piezon, EMS, Nyon, Switzerland) or EAP with and without chlorhexidine digluconate (EAP / EAP-CHX; Fig 2C / 2D; mid water and powder settings for 10 s, Air-Flow Master Piezon, EMS; Air-Flow Powder Plus 0.3% CHX, EMS). Each group, as well as an additional control group, consisted of 10 specimens per time point. All the treatment procedures were performed by well-experienced and calibrated periodontists (T.T.H. and R.C).

### Evaluation of reduction of bacterial counts

On once colonized and treated specimens, biofilm samples were collected from the surface and suspended in 0.9% w/v NaCl solution. After making a serial dilution each 25 μl was spread on Schaedler agar plates, incubated in anaerobic conditions and the total counts of colony forming units (CFU) were recorded. In addition the loads of selected periodontopathogens (*A*. *actinomycetemcomitans*, *P*. *gingivalis*, *T*. *forsythia*, *T*. *denticola*) were determined by real-time PCR [25].

### Evaluation of biofilm reformation

After treatment, specimens were exposed to UV for 30 min to kill contaminants (treatment could not be applied totally sterile; in part supply of tap water was needed). Then specimens were dipped into 25% inactivated human serum for 10 min again before they were placed back into tubes for renewed biofilm formation. After 3.5 days of incubation attached bacteria within biofilm were counted as described above.

### Evaluation of surface thickness and surface roughness

Specimens were characterized before and after treatment by measurements of specimen thickness at five pre-defined points as well as by profilometrical determination of the average surface roughness (Ra; μm) and the arithmetic mean height of the surface profile (Rz; μm) with a surface roughness meter (Perthometer S2; Mahr GmbH, Göttingen, Germany). Across each specimen, nine measurements were determined over a transverse length of L_t_ = 1.750 mm with a cut-off value of 0.25 mm. The specimens were turned 45° after three measurements (i.e., three measurements across the whole specimen at 0°, at 45°, and at 90°). From the nine Ra and Rz values, mean Ra and mean Rz value was calculated per specimen.

### Evaluation of attachment of periodontal ligament fibroblasts and release of interleukin-8

Human PDL fibroblasts were placed in T-25 cell culture flasks containing DMEM (Life Technologies / Invitrogen, Paisley, UK) with 10% fetal calf serum (FCS; Life Technologies / Invitrogen) to grow to confluence. At the starting of the experiments the fibroblasts were always in the third passage.

Once colonized and treated, specimens were exposed to UV for 30 min and placed into 24 well plates. Thereafter, PDL fibroblasts in DMEM with 10% FCS (both were added at a density of 10,000 cells / well and incubated at 37°C with 5% CO_2_. Culture medium was carefully exchanged after 40 h. At 72 h the fibroblasts were fixed and stained for adhesion experiments with DAPI staining (Roche Diagnostics GmbH, Mannheim, Germany). The attached fibroblasts were counted by using a fluorescent microscope (Olympus BX51, Tokyo, Japan). Three fields of 1 mm^2^ per specimen were counted and one mean value was calculated per specimen, which was used for analysis.

In addition, the amount of released interleukin (IL)-8 was determined in the cell cultivation media after 40 h when exchanging media by using commercially available enzyme-linked immunosorbent assay (ELISA) kits (R&D Systems Europe Ltd., Abingdon, UK) according to the manufacturer’s instruction.

### Repeated biofilm formation and retreatment

After exposing once colonized and treated specimens (incl. the untreated control) to UV for 30 min, biofilms were reformed within 3.5 days before reinstrumentation. The procedure was repeated 4x each after a 3.5 days biofilm formation, thus resulting in a dentine specimen instrumentation of totally five times. After five-fold treatment, the same evaluation as after one treatment cycle was performed, namely biofilm removal, recolonization, surface roughness and thickness as well the reattachment of PDL fibroblasts.

### Scanning electron microscopy photographs

Scanning electron microscopy (SEM) photographs were taken to visualize measurements of surface structure, removal and recolonization of microorganisms. Samples were fixed in 2% glutaraldehyde in cacodylate buffer for 30 min, washed twice with cacodylate buffer and dehydrated using a 10% graded ethanol series (10 min each concentration). Following critical point drying, samples were sputter-coated with gold and examined with a ZEISS LEO-1530 Gemini (Carl Zeiss NTS GmbH, Oberkochen, Germany) equipped with a field emission electron gun at 10 keV.

### Statistical analysis

All data are presented as mean and standard deviation (SD). Data were compared using a one-way analysis of variance (ANOVA) with post-hoc comparisons of groups using LSD corrections. A *p*-value of 0.05 was considered to be statistically significant. SPSS software (version 22.0) was used for statistical analysis.

## Results

### Removal and reformation of biofilms

The biofilm samples after one formation contained in mean 5.94±0.75 log10 CFU, the applied treatment modalities could reduce log10 CFU to a mean value of 2.83±1.66. The difference between the treatment modalities was up to 2.60 log10 CFU. Except for EAP-CHX group, which showed a reduced biofilm formation (4.94 log10 CFU) the recolonized biofilm contained 5.38–5.85 log10 CFU.

After five reformations of biofilm, the biofilm contained 7.52±0.68 log10 CFU, the applied treatment modalities could reduce log10 CFU to a mean value of 4.42±1.48. The difference between the treatment modalities was up to 3.03 log10 CFU. The thereafter reformed biofilms contained from 5.54 log10 CFU (EAP-CHX) up to 7.49 log10 CFU. Selected bacterial species were detectable. The used real-time PCR confirmed a relatively high quantity of *P*. *gingivalis* and *T*. *forsythia* in the model, *T*. *denticola* was present after repeated biofilm formation.

**Fig 3:**
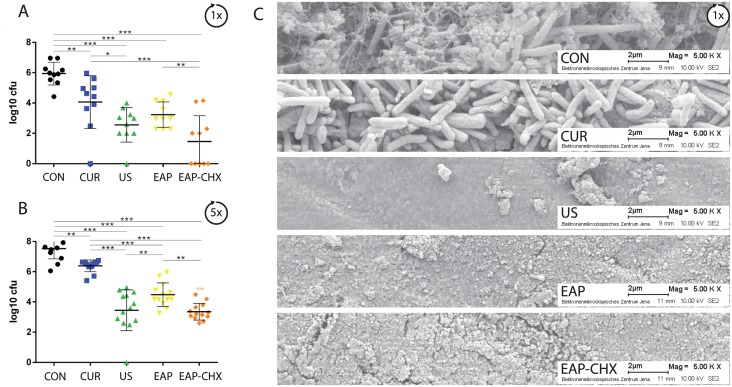
A-C. Biofilm removal. Graph depicting the mean colony forming units (log10 CFU, ±SD, n = 10; A, B) and SEM photographs (C) after one (A, C) and 5 times (B) biofilm formation (CON) and followed by instrumentation of the four different treatment methods (hand instrumentation (CUR), ultrasonication (US), erythritol air-polishing (EAP), and EAP with chlorhexidine digluconate (EAP-CHX). CFU were pronouncedly reduced by US and EAP-CHX (* p<0.05; ** p<0.01; *** p<0.001).

The removal of biofilm was expressed as bacterial reduction (log10 CFU reduction) after 1x and 5x instrumentation (Fig 3A+3B). After 1x treatment, significant differences were seen between all groups (p<0.001). All instrumentations significantly (p<0.01) reduced the log10 CFU compared with controls (Fig 3A). Applying EAP-CHX resulted in a bacterial reduction by 4 log10 CFU followed by EAP and US (each 3 log10 CFU), the difference of EAP-CHX was significant to CUR (p<0.001) and to EAP (p = 0.004). Similar results were found after 5x instrumentation (p<0.001 between all groups, p<0.001 each to controls; Fig 3B) with a reduction of 4 log10 CFU for EAP-CHX and US, followed by 3 log10 CFU reduction for EAP. The lowest reduction was found for both numbers of instrumentation after CUR (2 log10 CFU) being significantly less (p<0.001) than those by all other instrumentations.

**Fig 4:**
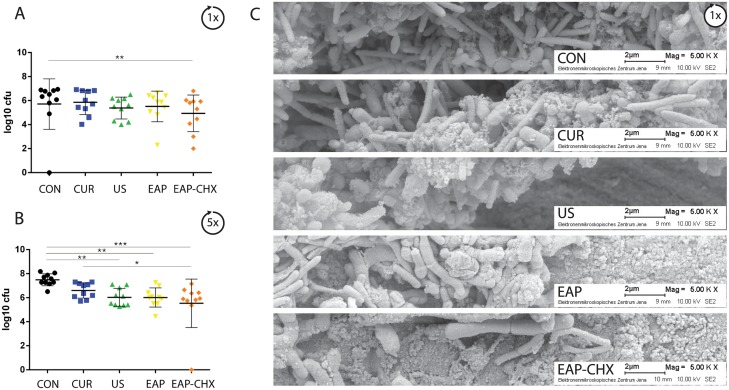
A-C. Reformation of biofilm. Graph presenting the mean colony forming units (log10 CFU, ±SD, n = 10; A, B) and SEM photographs (C) after one (A, C) and 5 times (B) biofilm formation (CON) followed by instrumentation of the four different treatment methods (hand instrumentation (CUR), ultrasonication (US), erythritol air-polishing (EAP), and EAP with chlorhexidine digluconate (EAP-CHX). All treatment modalities were followed by an additional biofilm formation cycle). Reformation of biofilm was delayed by US and EAP and EAP-CHX (* p<0.05; ** p<0.01; *** p<0.001).

The reformation of biofilms on the once treated surface differed only slightly (Fig 4A). Only surfaces exposed to EAP-CHX showed less biofilm formation compared to the control (p = 0.009). After five instrumentations, differences between the groups became significant (p<0.001). All treatments except for CUR negatively influenced the reformed biofilms (each p<0.01 compared to controls; Fig 4B). The lowest CFU were counted after EAP-CHX being significantly less not only to controls (p<0.001) but also to CUR (p<0.001) and to EAP only (p = 0.001).

SEM photographs taken after one treatment underline the findings in removal of biofilms (Fig 3C) and reformation of biofilms (Fig 4C).

**Fig 5:**
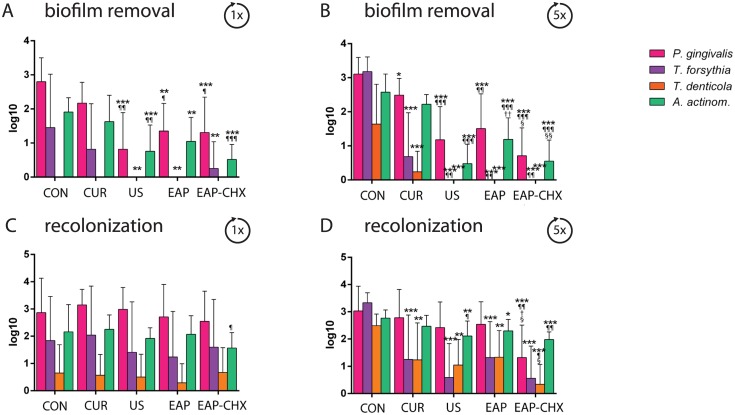
A-D. Biofilm removal and recolonization of selected bacterial species. Counts of selected bacterial species after biofilm removal and recolonization determined by real-time PCR. Graph presenting the mean log10 counts (±SD, n = 10) after one (A, C) and 5 times (B, D) biofilm formation and followed by instrumentation of the four different treatment methods (hand instrumentation (CUR), ultrasonication (US), erythritol air-polishing (EAP), EAP with chlorhexidine digluconate (EAP-CHX), and an untreated control (con) (A, B) as well as after an additional biofilm formation (C, D) (* p<0.05; ** p<0.01; *** p<0.001 compared to con, ^¶^p<0.05; ^¶¶^ p<0.01, ^¶¶¶^ p<0.001 compared to CUR, ^†^ p<0.05; ^††^ p<0.01 compared to US, ^§^ p<0.05; ^§§^ p<0.01 compared to EAP).

Single-species analysis (Fig 5A-5D) was in accordance with the results obtained for the total CFU counts. Instrumentations clearly reduced the counts of single species after one as well as after five applications. Inhibited recolonization was seen especially after five treatments. In general, EAP-CHX was most active in reducing counts after biofilm removal as well as in retarding recolonization.

### Substance loss and surface alterations

**Fig 6:**
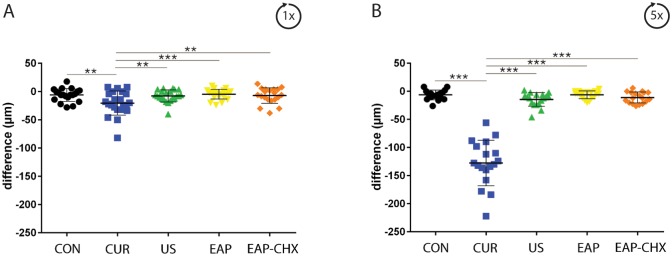
A+B. Tooth hard-substance-loss by different treatment methods. Graph presenting the thickness difference before and after one (A) and 5 times (B) instrumentation of the dentin specimens using four treatment methods (hand instrumentation (CUR), ultrasonication (US), (erythritol air-polishing (EAP), EAP with chlorhexidine digluconate (EAP-CHX)), and an untreated control (con). Instrumentation using CUR was related to significantly higher hard-substance loss compared to the other groups (** p<0.01; *** p<0.001).

Tooth substance loss measured as the difference of thickness before and after 1x and 5x instrumentation is presented in Fig 6A+6B. Even after one instrumentation cycle, instrumentation using curettes resulted in the highest tooth-substance-loss (21 μm±2 μm) compared to all the other groups (each p<0.01) and resulted in a highly significantly pronounced tooth-substance-loss of 128 μm±40 μm after 5x instrumentation (p < 0.001 to all groups).

**Fig 7:**
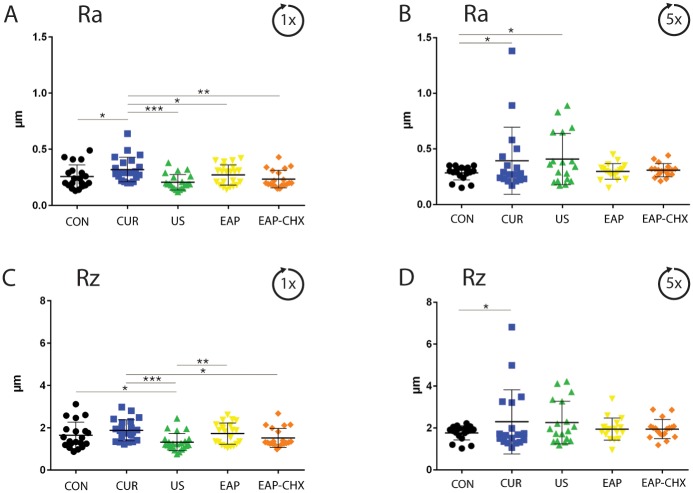
A-D. Tooth surface roughness. Graph depicting the average surface roughness Ra (A, B) and the arithmetic mean height of the surface profile Rz (C, D) after one (A, C) and 5 times (B, D) instrumentation of the dentin specimens using four treatment methods (hand instrumentation (CUR), ultrasonication (US), (erythritol air-polishing (EAP), EAP with chlorhexidine digluconate (EAP-CHX)), and an untreated control (con). Instrumentation using CUR and US resulted in an increased average surface roughness after 5 times instrumentation (* p<0.05; ** p<0.01; *** p<0.001).

Before instrumentation no difference of the average surface roughness (Ra) and of the arithmetic mean height of the surface profile (Rz) between the groups could be measured. After one treatment Ra and Rz differed between all groups (p = 0.002; p = 0.005). The followed post-hoc analysis revealed a higher Ra for CUR (p = 0.026) and a lower Rz for US (p = 0.037) each compared with the untreated control (Fig 7A+7C).

After five treatments Ra was higher after applying US (p = 0.037) and CUR (p = 0.030), Rz was higher after using CUR (p = 0.028) each compared with untreated controls (Fig 7B+7D).

#### Attachment of periodontal ligament fibroblasts

**Fig 8:**
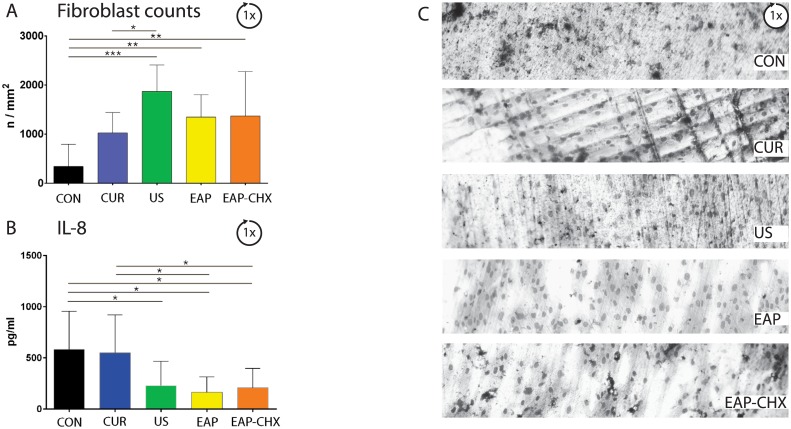
A-C. Attachment of periodontal ligament (PDL) fibroblasts after one treatment. Graphs presenting the mean attached PDL fibroblasts (A), release of IL-8 (B) and microscopic photographs (C) after one biofilm formation and one instrumentation of the dentin specimens using four treatment methods (hand instrumentation (CUR), ultrasonication (US), (erythritol air-polishing (EAP), EAP with chlorhexidine digluconate (EAP-CHX)), and an untreated control (con). IL-8 was measured in media after 40 h incubation. (* p<0.05; ** p<0.01; *** p<0.001).

**Fig 9:**
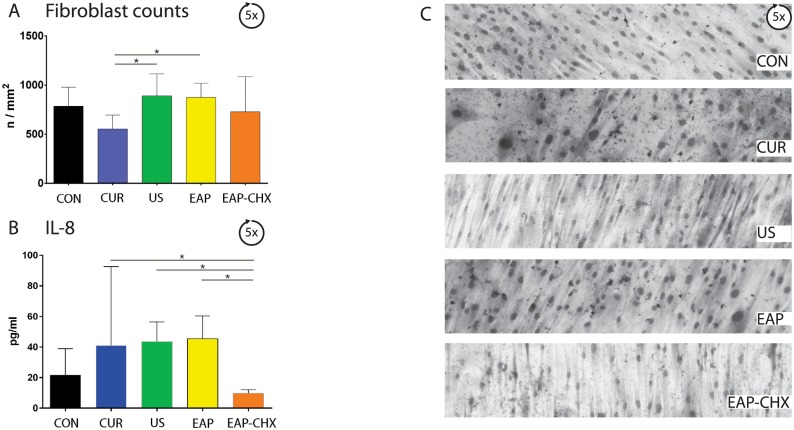
A-C. Attachment of periodontal ligament (PDL) fibroblasts after five treatments. Graphs presenting the mean attached PDL fibroblasts (A), release of IL-8 (B) and microscopic photographs (C) after five biofilm formations, five instrumentations of the dentin specimens using four treatment methods (hand instrumentation (CUR), ultrasonication (US), (erythritol air-polishing (EAP), EAP with chlorhexidine digluconate (EAP-CHX)), and an untreated control (con) and a complete biofilm removal. IL-8 was measured in media after 40 h incubation. (* p<0.05).

Attachment of PDL fibroblasts on the once treated surface differed significantly (p = 0.002). Except for CUR, counts of attached PDL fibroblasts were always higher compared with untreated control (p<0.01; Fig 8). The PDL fibroblast counts on the five times treated and thereafter cleaned test specimens were not clearly indicative (Fig 9). Surfaces exposed five times to US and to EAP attracted the highest numbers of PDL fibroblasts, the differences were significant to CUR (p = 0.017, p = 0.021).

Although less PDL fibroblasts were attached to the dentin specimens, a higher level of IL-8 after 1 instrumentation with CUR could be measured when compared to EAP, EAP-CHX (each p<0.05) and in the untreated control compared to US, EAP and EAP-CHX (each p<0.05). After five treatment cycles, less IL-8 was measured for EAP-CHX samples when compared to all other groups (p<0.05).

## Discussion

Clinical studies for treatment evaluation are designed to show inferiority, superiority or equivalence of a used method. Clinical trials are cost-intensive, ethical aspects need to be addressed and several individual factors might influence the outcome. On the contrary, in vitro studies are aimed to analyze a certain issue in a standardized manner thus guarantying reliable and reproducible results. They might reduce the number of patients to be included in clinical trials by focusing on the most promising methods and on the other hand may help to understand mechanism of action of a therapy. In the present in vitro study, a subgingival periodontal pocket model was established to analyze the effect of non-surgical periodontal therapy using different treatment modalities related to their capacity of biofilm removal, surface alterations, loss of tooth hard-substance, and attachment of PDL fibroblasts.

A number of in vitro studies showed that debridement by instrumentation in regular supportive periodontal therapy may result in surface alterations and hard-substance-loss [15,16,26,27]. However, to the best of our knowledge, these tooth hard-substance-alterations were never directly related to the treatment modality’s capacity of biofilm-removal using a multi-species biofilm model. Moreover, in the present study the biofilms have been repeatedly formed and traditional (CUR, US) as well as newly developed treatment modalities (EAP and EAP-CHX) were compared. Due to the significance of a simulation of subgingival water/powder swirls in a periodontal pocket resulting from the usage of the latter ones, we developed an artificial biofilm pocket model. Thus, the water/powder swirl resulting from the air-powder jet directing perpendicular to the root surface and the water-jet directing straight to the orifice of the pocket [18,19,20] and its influence on biofilm removal could be studied under in vitro conditions. Additionally, hand instrumentation using curettes and ultrasonication were performed under medium forces and with a working angle of 0° (US) to guarantee limited tooth substance loss [15,26,27] and to represent as much as possible daily practice.

In vitro study protocols only rarely include defined multi-species biofilms. Those biofilms were exposed to antibiotics [24,28], antiseptics, ozone [29], and photoactivated disinfection [29,30]. A multispecies biofilm consisting of 6 species grown on hydroxyapatite disks was treated with an ultrasonic scaler [31].

To the authors’ best knowledge, a repeated formation of multi-species biofilms has, so far, never been used to evaluate various nonsurgical periodontal treatment modalities. In the present experiment, a multi-species biofilm was formed several times directly on the same treated tooth surface. Microbial cultures confirmed the presence of the 12-species within the biofilms. However, before reformation the biofilms had to be exposed to UV to kill contaminants, a procedure that might have influenced the viability of the biofilms.

The combined determination of the different variables in this model resulted in the need for standardized test specimens with defined surfaces. This may reflect only partly the in vivo situation. However, in patients undergoing a continuous maintenance program, surfaces of teeth in the gingival region are characterized by a relatively flat surface topography due to repeated treatment procedures. Nevertheless, the model also allows to attach uneven parts of teeth to the plastic specimen holders and to perform experiments on biofilm formation as well as on applying alternative treatment procedures. It therefore might be adapted to evaluate oral hygiene procedures for daily use (e.g. rinsing with solutions, using tooth brushes with tooth pastes). In contrast to the present study set up, an application of sterile devices and materials would render an UV-exposure unnecessary.

Instrumentation by US as well as EAP / EAP-CHX was shown to be highly efficient, whereas biofilm removal performed by hand instruments using curettes was a less efficient treatment modality. The missing rinsing when using hand instruments however might have negatively influenced our results. Nevertheless, they are in agreement with clinical data for glycine air-polishing demonstrating higher cleaning efficacy when directly compared to hand instrumentation [32]. Moreover, comparison of glycine air-polishing and ultrasonic instrumentation revealed comparable results for patients in supportive periodontal therapy in terms of microbiological outcomes [20], also supporting our in vitro data. We considered the alternative use of erythritol powder instead of glycine to be equivalent since physical properties and abrasivity data for erythritol when compared to glycine were shown to be comparable [22]. On the contrary, erythritol was demonstrated to have antimicrobial activity and an inhibitory effect on biofilm formation [33]. Our results are in contrast to these findings, not showing additional antibacterial effects when applying erythritol air-polishing only. In a recent in vitro study, the antimicrobial and anti-biofilm activity of erythritol supplemented with chlorhexidine was higher than with glycine powder against single-species biofilms formed on titanium disks [34]. Using multi-species biofilms consisting of the most relevant bacteria associated with periodontitis, an additional antimicrobial activity after one and five treatments was confirmed in the present study. Chlorhexidine is widely used in periodontal therapy [35], but rinsing solutions are known to act predominantly in supragingival areas and can therefore mainly prevent from gingivitis [36]. By incorporation of chlorhexidine into a powder, access to the subgingival area might be provided through subgingival air-polishing. The removal of gingival crevicular fluid being rich of serum proteins [37] from the surface by the water/powder swirl may guarantee access to the root surface and avoid fast inactivation of chlorhexidine by serum components as recently shown [25]. Chlorhexidine is able to bind to root dentin surfaces providing a long-lasting post-antimicrobial activity [38,39].

Analyzing the surface properties after repeated US, a slight roughening was observed whereas hand instrumentation using curettes resulted in a high variability of results and demonstrated the difficulty of predictably achieve a standardized treatment outcome in terms of surface roughness. EAP and EAP-CHX seemed to be most favorable in terms of surface roughness. In general, low abrasive powders generate surfaces with low Ra values [40]. Even though after one exposure to US surfaces seemed to be smooth, conversely repeated treatments induced a higher roughness. It is difficult to transfer these data directly to an in vivo situation, since in our study the interval between two treatments was only 3.5 days and is therefore in contrast to the regular intervals of three to 12 months in supportive periodontal therapy. Controversial results were reported in comparable study settings: whereas in one study a treatment by ultrasonic curettes induced more surface roughness than hand curettes [41], other authors report an increasing Ra for curettes when comparing them to ultrasonication [42].

Tooth substance loss was determined by the difference of thickness of the test specimens. The measured tooth hard-substance-loss of 128 μm after 50 strokes in the present study goes in line with data of Zappa and co-workers [26] demonstrating tooth hard-substance-loss of 148 μm after 40 stokes using curettes with low force. In contrast, no significant substance loss could be measured by all other treatment modalities in the present study after 5x instrumentation. This is in accordance with data for glycine air-polishing showing low abrasivity for glycine [17,43] and for ultrasonication when applying an angulation of 0° and medium forces [27]. In contrast to these studies, dentin specimens were not statically instrumented but treated as in daily practice by dynamic movements.

Erythritol was recently introduced as valuable alternative to glycine air-polishing for plaque removal during supportive periodontal therapy due to its similar particle size distribution and abrasiveness [22]. Clinical data from patients treated for supportive periodontal therapy revealed comparable results after 3, 6, and 12 months when compared to hand instrumentation [21,22] and ultrasonication [23]. Erythritol air-polishing was shown to be safe, however not superior in terms of clinical and microbiological results [21,22,23]. However, due to limitations in terms of calculus removal, the use of air-polishing has to be restricted to supportive periodontal therapy.

Treated test specimens were exposed to PDL fibroblasts. One part of the experiment consisted of adding PDL fibroblasts to cleaned, five-fold treated test specimens with no residual biofilm. Thus, we could study selectively the influence of surface properties to the attachment (incl. IL-8 release) of fibroblasts. Differences were small and only surfaces treated with EAP and US attracted more fibroblasts than the untreated controls. Several in vitro-studies analyzed the influence of instrumentation on the attachment of PDL fibroblasts demonstrating inconsistent results [44,45]. In line with our results, Schwarz and co-workers observed an increased attachment of PDL fibroblasts to surfaces pre-treated with ultrasonic scalers than with SRP [46], however others observed less attachment after using conventional ultrasonic scaler [44]. The major difference was seen in IL-8 measurements after application of EAP-CHX. CHX having substantivity [47] may exert a long-lasting activity. In a biofilm-epithelial cell model, application of chlorhexidine reduced mRNA and protein expression of IL-8 [48]. Our results are in line with this finding supporting an anti-inflammatory activity of CHX.

On one-fold treated test specimens the biofilm was not removed, but the bacteria were killed by UV. The remaining bacterial compounds (e.g. lipopolysaccharides (LPS)) may have interfered with PDL fibroblasts. LPS was shown to cause reduction of cell orientation [49]. Differences in the numbers of attached PDL fibroblasts as well as of the measured IL-8 were more remarkable after one treatment, which underlines an overwhelming role of the remnants of the biofilm.

## Conclusion

A biofilm model simulating a periodontal pocket combined with interchangeable placements of test specimens with biofilms enables the evaluation of different repeated applied non-surgical treatment modalities both on biofilm removal and surface alterations. Moreover, it is suitable for follow-up investigations, e.g. reformation of biofilms, interaction with host cells.

When considering the relation between surface disinfection and surface-alterations, it must be concluded that in particular after hand instrumentation using curettes the outcome is not favorable in comparison with the other treatment modalities. Air-polishing with erythritol resulting in no substance loss and a smooth surface combined with nearly no residual biofilm seems to be a promising alternative for patients in need of intensive supportive periodontal therapy. Supplementation of erythritol with chlorhexidine results in an increase in antibacterial activity and should be further analyzed in preclinical and clinical trials.

## Supporting Information

S1 File Supporting data to the Figs 3–9. The single data or means and SD for Figs 3–9 are presented.
(PDF)
