# Supplementary material for: A Biofilm Pocket Model to Evaluate Different Non-Surgical Periodontal Treatment Modalities in Terms of Biofilm Removal and Reformation, Surface Alterations and Attachment of Periodontal Ligament Fibroblasts
Source: PLoS One. 2015 Jun 29;10(6):e0131056. doi: 10.1371/journal.pone.0131056 (PMC4486723; doi:10.1371/journal.pone.0131056)
Supplement: S1 File — The single data or means and SD for Figs 3–9 are presented. (PDF) [file pone.0131056.s001.pdf]

Data Fig. 3 A-C. Biofilm removal.

Colony forming units (single values, log10) after one and 5 times biofilm formation (CON) and followed by instrumentation of the four different treatment methods (hand instrumentation (CUR), ultrasonication (US), erythritol air-polishing (EAP), and EAP with chlorhexidine digluconate (EAP-CHX).

After one treatment

Sample	Con	CUR	US	APE	APE-CHX
1.	5.98	4.43	2.60	4.20	0.00
2.	5.92	5.97	3.23	2.30	4.16
3.	5.54	5.61	3.00	3.00	2.00
4.	4.42	2.48	0.00	2.30	2.00
5.	6.17	3.91	2.00	2.95	2.30
6.	5.87	0.00	2.00	2.95	0.00
7.	6.25	4.96	3.04	4.55	0.00
8.	5.34	4.63	3.68	4.13	0.00
9.	6.96	5.00	4.00	3.59	0.00
10.	6.96	3.63	2.00	2.30	4.10

After five treatments

Sample	Con	CUR	US	APE	APE-CHX
1.	7.58	5.41	3.34	5.15	3.38
2.	7.26	6.32	3.60	5.96	2.95
3.	7.93	6.75	2.60	3.26	3.15
4.	6.89	6.37	2.90	4.19	3.32
5.	6.06	6.55	4.66	4.62	3.80
6.	6.49	5.72	3.89	4.18	4.20
7.	8.09	6.63	4.95	3.58	3.04
8.	8.02	6.64	4.36	5.74	4.49
9.	8.24	6.33	4.43	4.49	3.20
10.	8.05	6.58	4.79	4.19	2.70
11.	7.46	6.60	2.78	4.78	2.60
12.	8.08	6.49	2.48	4.15	3.08

Data Fig. 4 A-C. Reformation of biofilm

Colony forming units (single values, log10) after one and 5 times biofilm formation (CON) followed by instrumentation of the four different treatment methods (hand instrumentation (CUR), ultrasonication (US), erythritol air-polishing (EAP), and EAP with chlorhexidine digluconate (EAP-CHX). All treatment modalities were followed by an additional biofilm formation cycle).

After one treatment

Sample	Con	CUR	US	APE	APE-CHX
1.	4.90	6.84	5.32	6.65	5.93
2.	6.95	6.03	5.51	5.88	6.80
3.	6.52	6.92	6.01	5.45	5.82
4.	6.84	4.60	4.00	6.40	5.30
5.	6.78	6.77	6.50	6.41	6.03
6.	6.89	6.83	4.30	5.88	4.48
7.	5.80	4.03	5.61	2.30	2.00
8.		5.45	4.21	4.85	3.00
9.	6.08	5.72	6.20	5.08	4.09
10.	6.34	5.34	6.16	6.24	5.93

After five treatments

Sample	Con	CUR	US	APE	APE-CHX
1.	7.88	6.20	6.13	6.08	5.75
2.	7.28	6.10	5.41	6.05	6.42
3.	6.51	7.00	5.53	7.00	0.00
4.	7.58	5.79	5.36	5.51	5.94
5.	8.06	7.30	6.83	6.41	6.37
6.	7.26	7.18	7.08	4.44	7.15
7.	7.71	7.08	7.11	7.26	6.65
8.	8.19	6.36	5.41	5.90	5.90
9.	7.15	7.30	6.20	5.99	5.82
10.	7.28	5.72	5.26	5.51	5.36

Data Fig. 5 A-D. Biofilm removal and recolonization of selected bacterial species.

Counts of selected bacterial species after biofilm removal and recolonization**.** Mean log10 counts (±SD, n=10) after one and 5 times biofilm formation and followed by instrumentation of the four different treatment methods (hand instrumentation (CUR), ultrasonication (US), erythritol air-polishing (EAP), EAP with chlorhexidine digluconate (EAP-CHX), and an untreated control (con) as well as after an additional biofilm formation

Counts after one treatment

	P. gingivalis		***T. forsythia***		***T. denticola***		***A. actinom.***	
	Mean	SD	Mean	SD	Mean	SD	Mean	SD
**Con**	4.80	0.70	3.45	1.57	1.89	0.30	3.91	0.42
**CUR**	4.17	0.61	1.52	2.25	0.20	0.63	3.63	0.77
**US**	1.12	0.89	0.00	0.00	0.00	0.00	1.66	0.95
**APE**	2.25	1.27	0.00	0.00	0.00	0.00	2.15	0.99
**APE-CHX**	1.71	1.13	0.75	1.08	0.42	0.81	1.07	0.73

Recolonization after one treatment

	P. gingivalis		***T. forsythia***		***T. denticola***		***A. actinom.***	
	Mean	SD	Mean	SD	Mean	SD	Mean	SD
**Con**	4.87	1.26	3.84	1.62	2.65	1.04	4.16	1.00
**CUR**	5.15	0.57	3.74	2.20	2.07	1.22	4.25	0.53
**US**	4.99	0.80	3.41	1.85	2.51	0.84	3.92	0.39
**APE**	4.61	1.45	3.15	1.77	1.94	1.32	3.96	0.94
**APE-CHX**	3.95	1.33	3.00	1.84	2.06	1.28	2.97	1.23

Counts after removal after five treatments

	P. gingivalis		***T. forsythia***		***T. denticola***		***A. actinom.***	
	Mean	SD	Mean	SD	Mean	SD	Mean	SD
**Con**	5.11	0.49	5.18	0.43	3.64	1.17	4.58	0.53
**CUR**	4.48	0.49	1.91	1.82	1.28	1.31	4.22	0.29
**US**	1.94	1.31	0.00	0.00	0.00	0.00	0.94	1.07
**APE**	2.28	1.41	0.00	0.00	0.00	0.00	2.03	0.96
**APE-CHX**	1.32	1.23	0.00	0.00	0.00	0.00	1.24	0.99

Recolonization after five treatments

	P. gingivalis		***T. forsythia***		***T. denticola***		***A. actinom.***	
	Mean	SD	Mean	SD	Mean	SD	Mean	SD
**Con**	5.03	0.91	5.33	0.37	4.49	0.43	4.77	0.30
**CUR**	4.78	1.04	3.25	1.63	3.24	1.35	4.47	0.40
**US**	4.42	0.94	2.34	1.43	2.85	1.23	4.11	0.55
**APE**	4.54	0.83	1.71	2.17	2.73	1.92	4.30	0.42
**APE-CHX**	2.92	1.81	1.16	1.96	0.94	1.52	3.78	0.69

Data Fig. 6 A+B. Tooth hard-substance-loss by different treatment methods.

Thickness difference (µm) before and after one and 5 times instrumentation of the dentin specimens using four treatment methods (hand instrumentation (CUR), ultrasonication (US), (erythritol air-polishing (EAP), EAP with chlorhexidine digluconate (EAP-CHX)), and an untreated control (con).

After one treatment

**Sample**	**Con**	**CUR**	**US**	**APE**	**APE-CHX**
1.	-10.	-24.	-2.	-4.	2.
2.	-28.	-46.	-18.	-6.	-10.
3.	-26.	-28.	4.	-10.	-30.
4.	2.	-14.	-20.	-2.	-10.
5.	-6.	-50.	-40.	2.	-6.
6.	6.	-4.	-8.	-2.	6.
7.	18.	8.	6.	-2.	-16.
8.	0.	-22.	4.	2.	-8.
9.	-8.	-4.	-8.	10.	14.
10.	-6.	6.	0.	6.	4.
11.	0.	0.	-10.	-2.	8.
12.	-6.	-18.	0.	-2.	-14.
13.	-6.	-14.	-16.	-20.	-14.
14.	-18.	-20.	-6.	-2.	4.
15.	-2.	-32.	-12.	-20.	4.
16.	6.	-82.	4.	6.	-2.
17.	-4.	0.	-12.	-6.	-38.
18.	-14.	8.	-8.	-2.	0.
19.	4.	-32.	-6.	-14.	-30.
20.	-22.	-24.	6.	-24.	-6.
21.		-34.	-14.	-8.	
22.		-24.	-12.	0.	

After five treatments

Sample	Con	CUR	US	APE	APE-CHX
1.	-10.	-90.	-22.	-16.	-24.
2.	-14.	-98.	-8.	4.	-22.
3.	-16.	-132.	-12.	-10.	-12.
4.	0.	-78.	2.	-2.	0.
5.	8.	-88.	-8.	0.	0.
6.	-8.	-112.	-10.	-2.	-2.
7.	-4.	-178.	-2.	0.	-2.
8.	-6.	-184.	-12.	-2.	-12.
9.	-26.	-158.	-34.	-20.	-20.
10.	-14.	-134.	-6.	-4.	-22.
11.	8.	-128.	-2.	-18.	-14.
12.	-10.	-130.	-24.	-4.	-8.
13.	-4.	-56.	-20.	-12.	-2.
14.	-2.	-222.	-10.	-4.	6.
15.	-8.	-124.	-46.	0.	-16.
16.	2.	-140.	-24.	-6.	-26.
17.	-4.	-136.	-16.	-12.	-4.
18.		-110.	-6.	-4.	-18.

Data Fig. 7 A-D. Tooth surface roughness.

Average surface roughness Ra and the arithmetic mean height of the surface profile Rz (µm) after one and 5 times instrumentation of the dentin specimens using four treatment methods (hand instrumentation (CUR), ultrasonication (US), (erythritol air-polishing (EAP), EAP with chlorhexidine digluconate (EAP-CHX)), and an untreated control (con).

Ra after one treatment

Sample	Con	CUR	US	APE	APE-CHX
1.	0.24	0.20	0.15	0.22	0.18
2.	0.26	0.49	0.19	0.21	0.20
3.	0.18	0.30	0.18	0.31	0.20
4.	0.31	0.40	0.21	0.34	0.31
5.	0.29	0.34	0.32	0.40	0.33
6.	0.43	0.29	0.38	0.42	0.35
7.	0.13	0.21	0.15	0.31	0.18
8.	0.20	0.29	0.16	0.30	0.19
9.	0.15	0.22	0.19	0.21	0.19
10.	0.18	0.20	0.12	0.35	0.18
11.	0.24	0.25	0.29	0.39	0.22
12.	0.20	0.43	0.31	0.40	0.23
13.	0.20	0.27	0.14	0.16	0.17
14.	0.20	0.29	0.18	0.18	0.29
15.	0.49	0.64	0.14	0.17	0.34
16.	0.41	0.28	0.25	0.30	0.43
17.	0.41	0.45	0.26	0.30	0.19
18.	0.29	0.38	0.20	0.30	0.15
19.	0.17	0.21	0.15	0.21	0.20
20.	0.14	0.33	0.19	0.18	0.15
21.		0.26	0.18	0.16	
22.		0.29	0.19	0.14	

Rz after one treatment

Sample	Con	CUR	US	APE	APE-CHX
1.	1.53	1.22	0.98	1.32	1.22
2.	1.83	2.81	1.32	1.35	1.32
3.	1.19	1.66	1.21	2.06	1.35
4.	1.93	2.31	1.36	2.16	1.99
5.	1.84	2.04	1.95	2.38	2.14
6.	2.61	2.00	2.45	2.62	2.15
7.	0.87	1.34	1.05	1.98	1.31
8.	1.33	1.86	1.08	1.90	1.30
9.	1.06	1.32	1.25	1.54	1.25
10.	1.26	1.31	0.85	2.12	1.28
11.	1.56	1.57	1.86	2.35	1.40
12.	1.25	2.42	1.98	2.40	1.36
13.	1.36	1.33	0.76	1.11	1.20
14.	1.29	1.94	1.15	1.13	1.82
15.	3.12	2.98	0.94	1.23	2.12
16.	2.58	1.74	1.46	1.88	2.68
17.	2.47	2.49	1.47	1.87	1.25
18.	1.76	2.26	1.18	1.82	0.99
19.	1.09	1.41	1.05	1.35	1.25
20.	0.98	1.89	1.27	1.18	1.09
21.		1.73	1.19	1.14	
22.		1.78	1.23	1.05	

Ra after five treatment

Sample	Con	CUR	US	APE	APE-CHX
19.	0.26	0.58	0.18	0.32	0.32
20.	0.29	1.38	0.28	0.36	0.41
21.	0.35	0.50	0.65	0.30	0.27
22.	0.29	0.21	0.17	0.32	0.32
23.	0.35	0.28	0.21	0.33	0.30
24.	0.28	0.23	0.83	0.23	0.44
25.	0.33	0.29	0.39	0.31	0.31
26.	0.30	0.27	0.20	0.27	0.30
27.	0.35	0.22	0.69	0.29	0.31
28.	0.32	0.35	0.34	0.27	0.27
29.	0.18	0.21	0.23	0.22	0.21
30.	0.15	0.23	0.38	0.15	0.23
31.	0.32	0.27	0.21	0.35	0.32
32.	0.29	0.89	0.65	0.29	0.38
33.	0.30	0.24	0.89	0.45	0.31
34.	0.33	0.33	0.39	0.22	0.24
35.	0.24	0.17	0.36	0.27	0.34
36.	0.17	0.43	0.29	0.40	0.27

Rz after five treatments

Sample	Con	CUR	US	APE	APE-CHX
1.	1.63	3.48	1.32	2.02	2.08
2.	1.85	6.81	1.73	2.46	2.55
3.	1.97	3.25	3.27	1.89	1.72
4.	1.76	1.28	1.18	1.97	1.98
5.	2.07	1.65	1.49	2.05	2.01
6.	1.86	1.50	4.12	1.47	2.86
7.	1.97	1.72	2.21	2.02	2.02
8.	1.88	1.35	1.34	1.79	1.66
9.	2.22	1.27	3.72	1.95	1.84
10.	1.91	1.98	1.86	1.69	1.73
11.	1.20	1.32	1.38	1.48	1.18
12.	1.02	1.43	1.96	0.95	1.36
13.	2.05	1.68	1.38	2.24	1.95
14.	1.78	4.99	3.35	1.81	2.88
15.	1.92	1.52	4.22	2.59	2.00
16.	2.11	1.74	2.30	1.44	1.57
17.	1.50	1.06	2.07	1.81	2.07
18.	1.14	3.21	1.71	3.39	1.55

Data Fig. 8 A-C. Attachment of periodontal ligament (PDL) fibroblasts after one treatment. Mean attached PDL fibroblasts, release of IL-8 and SEM photographs after one biofilm formation and one instrumentation of the dentin specimens using four treatment methods (hand instrumentation (CUR), ultrasonication (US), (erythritol air-polishing (EAP), EAP with chlorhexidine digluconate (EAP-CHX)), and an untreated control (con). IL-8 was measured in media after 40 h incubation.

	**Counts/mm^2**		**IL-8**	
	Mean	SD	Mean	SD
**Con**	342.67	453.07	582.48	375.19
**CUR**	1024.00	416.26	549.03	372.13
**US**	1869.17	538.85	229.68	239.29
**APE**	1346.67	455.5	165.37	150.91
**APE-CHX**	1369.17	905.1	208.03	188.09

Data Fig. 9 A-C. Attachment of periodontal ligament (PDL) fibroblasts after five treatments.

Mean attached PDL fibroblasts, release of IL-8 after five biofilm formations, five instrumentations of the dentin specimens using four treatment methods (hand instrumentation (CUR), ultrasonication (US), (erythritol air-polishing (EAP), EAP with chlorhexidine digluconate (EAP-CHX)), and an untreated control (con) and a complete biofilm removal. IL-8 was measured in media after 40 h incubation.

	**Counts/mm^2**		**IL-8**	
	Mean	SD	Mean	SD
**Con**	784.17	195.2	21.59	17.44
**CUR**	553.67	139.99	41.08	51.57
**US**	889.00	223.66	43.65	12.88
**APE**	874.33	143.74	45.32	15.01
**APE-CHX**	727.67	357.74	9.82	2.21
